# Eutrema *EsMYB90* Gene Improves Growth and Antioxidant Capacity of Transgenic Wheat Under Salinity Stress

**DOI:** 10.3389/fpls.2022.856163

**Published:** 2022-04-29

**Authors:** Chuanshun Li, Yaoyao Zhao, Yuting Qi, Chonghao Duan, Hengyang Zhang, Quan Zhang

**Affiliations:** Shandong Provincial Key Laboratory of Plant Stress Research, College of Life Science, Shandong Normal University, Jinan, China

**Keywords:** *EsMYB90* gene, *Eutrema salsugineum*, ectopic overexpression, salt stress, antioxidant capacity, *Triticum aestivum*, RNA-seq

## Abstract

The ectopic expression of the *EsMYB90* transcription factor gene from halophytic *Eutrema salsugineum* has been reported to enhance the level of anthocyanin and other flavonoid metabolites in transgenic tobacco. In this study, the wheat JW1 overexpressing *EsMYB90* showed longer roots and higher fresh weight than that in wild type (WT) under salt stress. In addition, the transgenic wheat plants displayed significantly higher peroxidase (POD) and glutathione S-transferase (GST) activity, as well as markedly lower malondialdehyde (MDA) content than that of the WT during salt stress conditions. The analysis of histochemical staining and H_2_O_2_ level indicated that the accumulation of reactive oxygen species (ROS) was significantly lower in the roots of transgenic wheat plants compared to the WT under salt stress. Transcriptome analysis revealed that the *EsMYB90* gene affected the expression of considerable amounts of stress-related genes that were involved in phenylpropanoid biosynthesis and antioxidant activity in transgenic plants subjected to NaCl treatment. Importantly, the significantly upregulated expression genes in transgenic wheat under salt stress were mainly associated with the antioxidative enzymes POD and GST encoding genes compared with the WT. Furthermore, *EsMYB90* is suggested to bind with the MYB-binding elements of *pTaANS2* and *pTaDFR1* by dual luciferase assay, to activate the transcription of *TaANS2* and *TaDFR1* genes that are encoding key enzymes of anthocyanin biosynthesis in transgenic wheat plants. All the results indicated that, under salt stress, the *EsMYB90* gene plays a crucial role in preventing wheat seedlings from oxidative stress damage *via* enhancing the accumulation of non-enzymatic flavonoids and activities of antioxidative enzymes, which suggested that *EsMYB90* is an ideal candidate gene for the genetic engineering of crops.

## Introduction

Soil salinity is a worldwide problem that adversely affects plant growth and productivity by afflicting virtually every aspect of plant physiology and metabolism (Chinnusamy et al., 2005; Zhao et al., 2020). Wheat (*Triticum aestivum* L.) is a major cereal grown throughout the world, and its grain yield is significantly restrained by salinity stress (Zhang et al., 2014; Luo et al., 2019). Therefore, further research is needed to understand the molecular mechanisms underlying the response and tolerance of plants to salt stress, and obtain key candidate genes for the genetic engineering of crops, ultimately leading to improving the salt tolerance of wheat.

The exposure of plants to salt stress environments will disrupt many physiological and biochemical functions in plant cells (Sairam and Tyagi, 2004). Soil salinity stress firstly causes osmotic stress including loss of turgidity in plant cells produced by the disruption of the water potential and decreased size of stomatal apertures to balance the water pressure (Munns and Tester, 2008). Subsequently, the plant tissues, especially the above-ground parts, accumulate excessive toxic sodium, which results in an overproduction of reactive oxygen species (ROS) under saline stress (Larcher, 2003; Munns and Tester, 2008). Additionally, the oxidative damage of plant cells caused by excessive ROS accumulation will eventually trigger photosynthesis inhibition and metabolic damage, further impair fertility and growth, and provoke early senescence in plants (Larcher, 2003). Therefore, plants have evolved a series of complex enzymatic and non-enzymatic antioxidant defense mechanisms to maintain cell ROS homeostasis and minimize oxidative damage (De Azevedo Neto et al., 2006; Hernández et al., 2009). The enhanced activity of ROS-scavenging enzymes, such as superoxide dismutase (SOD), catalase (CAT), peroxidase (POD), ascorbate peroxidase (APX), and glutathione S-transferase (GST) is the most common mechanism for detoxifying ROS generated during stress response (Latef and Chaoxing, 2011; Islam et al., 2018). Meanwhile, flavonoids acting as non-enzymatic scavengers of ROS, can be induced by various biotic and abiotic stresses, and protect plants against damages from environmental stimuli (Dixon and Paiva, 1995; Agati et al., 2013; Qi et al., 2021).

The various types of transcription factors (TFs) are performed as nodes involved in a complicated signaling transduction network, and play central roles in the adaptation of plants to environmental stresses, such as salt and drought stresses (Stracke et al., 2001; Verslues et al., 2006; Dubos et al., 2010; Zhang et al., 2012). MYB (v-myb avian myeloblastosis viral oncogene homolog) TFs in plants are characterized by the presence of a highly conserved MYB DNA-binding domain that typically contains one to four imperfect repeats (Li et al., 2015). It is reported that MYB proteins are involved in the regulation of numerous stress-related genes directly or indirectly in response to abiotic stresses (Valliyodan and Nguyen, 2006; Shinozaki and Yamaguchi-Shinozaki, 2007; Li et al., 2015). The downstream genes of MYB protein have been found to contain RD22, SOSs, PP2Cs, ROS scavenging proteins, abscisic acid (ABA) synthesis proteins, cell expansion, and cuticle metabolism proteins, as well as other stress-induced proteins (Cominelli et al., 2008; Jung et al., 2008; Seo et al., 2009; Dai et al., 2012; Qin et al., 2012; Kim et al., 2013).

The R2R3-MYB subfamily is the largest group in the plant MYB family and is documented by more than 126 members in *Arabidopsis* and about 109 members in rice (Yanhui et al., 2006). The expansion of R2R3-MYB proteins indicates that they may play essential roles in plant-specific processes including the regulation of primary and secondary metabolism, seed and floral development, cell fate and identity, and defense and stress responses in plants (Dubos et al., 2010). It has been demonstrated that a specific calmodulin isoform mediates salt-induced Ca^2+^ signaling through the activation of *AtMYB2* to regulate salt responsive genes, and conferred tolerance of *Arabidopsis* plants to salt stress (Abe et al., 2003; Yoo et al., 2005). In rice, the overexpression of the *OsMYB2* gene led to a higher accumulation of soluble sugars and proline and less accumulation of H_2_O_2_ and malondialdehyde (MDA), as well as the upregulation of genes responsible for proline synthesis under salt stress, indicating effective osmoregulation and lower oxidative damage in plants under salt stress (Yang et al., 2012). AtMYB20 improves the salt resistance of transgenic overexpressing *Arabidopsis* plants by binding to the promoter of *ABA insensitive1* (*ABI1*) and type 2C serine/threonine protein phosphatase A (*PP2CA*) encoding genes, which are negative regulators of ABA signaling (Cui et al., 2013). The expression of *ABI1*, *ABI2*, and *AtPP2CA* genes was suppressed in *AtMYB20*-overexpressing lines but induced in *AtMYB20*-repression lines when plants were subjected to NaCl treatment (Cui et al., 2013). The *Arabidopsis bos1* mutant is hypersensitive to salt, drought, and oxidative stress; thus, BOS1/MYB108 is suggested as a positive regulator of the abiotic stress responses (Mengiste et al., 2003). In wheat, *the TaMYB33* gene was induced by NaCl, polyethylene glycol (PEG), and ABA treatments, and the putative ABA-responsive element (ABRE), MYB, and other abiotic stress related *cis*-elements are present in its promoter sequence (Qin et al., 2012). Ectopic overexpression of *TaMYB33* in *Arabidopsis* remarkably enhanced its tolerance to NaCl and drought stresses. Additionally, the expression of *AtP5CS* and *AtZAT12* genes as well as the proline content and ascorbate peroxidase (ASA-POD) activity were improved in *TaMYB33* overexpression lines, illustrating that *TaMYB33* enhanced salt and drought tolerance partially through the superior ability of osmotic balance reconstruction and ROS detoxification (Qin et al., 2012). TaMYB73, an R2R3 type MYB protein with some stress-, ABA-, and gibberellins (GA)-responsive *cis*-elements present in its promoter region, and its overexpression in *Arabidopsis* enhanced the tolerance of plants to NaCl and had superior germination ability under NaCl and ABA treatments (He et al., 2012). TaMYB73 could bind to the promoter sequences of *AtCBF3* and *AtABF3* genes and enhanced the expression of many stress-signaling genes, such as *AtCBF3* and *AtABF3*, as well as downstream responsive genes, *AtRD29A* and *AtRD29B*, in these overexpression lines, thus suggesting TaMYB73 conferred the salt tolerance of *Arabidopsis* by regulating the expression of stress related genes (He et al., 2012). However, *Arabidopsis* AtMYB73 was reported to act as a negative regulator of salt response *via* suppression of salt overly sensitive (*SOS*) genes, and the knocking out lines of the *AtMYB73* gene exhibited higher survival rates and markedly enhanced tolerance to salinity stress, compared to the wild type (WT) (Kim et al., 2013). Besides, the ectopic expression of an R2R3 MYB gene *TaPIMP* from wheat in tobacco was found to confer increased resistance to salt and drought stresses (Liu et al., 2011). In saline and drought conditions, *TaMYBsdu1* exhibited a stronger expression in salt-tolerant wheat cultivar than that in salt-sensitive cultivar, and it is inferred to be involved in wheat adaptation to salt and drought environments (Rahaie et al., 2010). In addition, OsMPS, an R2R3-type MYB transcription factor of rice, played important role in the response to abiotic stresses by directly modulating the expansin and endoglucanase genes (Schmidt et al., 2013). The expression of *OsMPS* was induced by salinity stress, but its overexpression reduced plant growth under normal conditions. Expression profiling revealed that OsMPS negatively regulated the expression of *EXPANSIN* (*EXP*), cell-wall biosynthesis as well as phytohormone signaling genes (Schmidt et al., 2013). Furthermore, OsMPS was a direct upstream regulator of some expansin genes (*OsEXPA4*, *OsEXPA8*, *OsEXPB2*, *OsEXPB3*, *and OsEXPB6*) and endoglucanase genes *OsGLU5* and *OsGLU14*. In addition, brassinolide (BR) biosynthesis and signaling genes were found to be downregulated upon the overexpression of *OsMPS*; thus, it was suggested that an integrative function in the cross-talk between phytohormones and the environment regulates adaptive growth (Schmidt et al., 2013).

Anthocyanin as a kind of flavonoid pigment can protect plants from abiotic stresses, and the regulation of anthocyanin biosynthesis displays a cross-talk with the diverse abiotic stresses (Dixon and Paiva, 1995; Agati et al., 2013). Among these, MYB proteins play essential roles by modulating the expression of a large number of flavonoid biosynthetic genes (Ali, 2014). It is now clear that the R2R3-MYB proteins, such as *Arabidopsis* production of anthocyanin pigment 1–4 (PAP1–4) namely AtMYB75, AtMYB90, AtMYB113, and AtMYB114, *Arabidopsis* TT2 (AtMYB123), as well as grape VvMYBPA2 and VvMYBA2, regulate anthocyanin and proanthocyanidin pathways (Paz-Ares et al., 1987; Borevitz et al., 2000; Nesi et al., 2001; Gonzalez et al., 2008; Heppel et al., 2013). However, AtMYB7 functions as a repressor of flavonol biosynthesis, which is downregulated by AtMYB4. AtMYB4 and AtMYB32, characterized as the closest homologs of AtMYB7 in *Arabidopsis*, were described as repressors of different branches of phenylpropanoid metabolism (Fornalé et al., 2014). In addition, some flavonoid biosynthetic genes are even the direct targets of MYB proteins in response to abiotic stresses. For example, MYB112, upregulated by salinity, could bind with the promoters of *MYB7* and *MYB32*, and the MYB112 was required for promoting the anthocyanin accumulation upon salt and high light stress (Lotkowska et al., 2015).

Although some MYB family members have been identified for their function in stress tolerance and/or flavonoid biosynthesis, the salt tolerance of the MYBs from halophytes still needs to be explored, which will be significant for the academic and applied studies to improve the stress tolerance of crops. In our previous studies, an R2R3 MYB *EsMYB90* gene identified from halophytic *Eutrema salsugineum* (salt cress) played a critical regulating role in promoting many flavonoid compounds biosynthesis in transgenic tobacco, a dicotyledon model (Qi et al., 2020, 2021). Wheat is a major monocotyledon grain crop grown worldwide, but it is a glycophyte and cannot cope with high salinity (Xue et al., 2004; Munns and Tester, 2008). Given the crucial function of *Eutrema EsMYB90* in upregulating many flavonoid compounds content in transgenic tobacco, as well as the notable contribution of flavonoids in the stress tolerance of plants, we explore further its function in the salt tolerance breeding of wheat by genetic manipulation.

In this study, we report on the function of *EsMYB90* by its overexpression in wheat (*Triticum aestivum* L.) and revealed that *EsMYB90* overexpression results in longer roots, greater fresh weight, and higher flavonoid content, as well as enhanced activity of antioxidant enzymes, and an alleviated ROS damage when the plants were subjected to 200 mM NaCl stress. Transcriptome and quantitative RT-PCR documented that salinity stress affected the expression of plentiful antioxidant and stress-related genes including *PODs* and *GSTs* genes, as well as the encoding genes of anthocyanin synthase (ANS), dihydroflavonol 4-reductase (DFR), and glucan endo-1, 3-β-glucosidase (GEBG) genes. Therefore, the key function of the *EsMYB90* gene is suggested in improving antioxidant capacity to detoxify ROS in transgenic wheat plants subjected to salt stress. This research will help to understand the regulating mechanisms of the *EsMYB90* gene involved in the salt tolerance of plants, and indicate that *EsMYB90* is an ideal candidate gene for the genetic engineering of crops.

## Materials and Methods

### Generation of Transgenic Wheat Plants

*EsMYB90* gene sequence was amplified using KOD-Plus-Neo DNA polymerase (Toyobo, Japan) and linked to a 3 × Flag tag. The *3* × *Flag-EsMYB90* fragments were cloned into the *LGYOE3* vector under the control of a ubiquitin promoter. The constructs of *3* × *Flag-EsMYB90-LGYOE3* were introduced into *Agrobacterium tumefaciens* strain EHA105, and the immature embryos of wheat (JW1) were infected with the EHA105 carrying the overexpression vector according to the method described by Zhang et al. (2018). The grinding leaf of the transgenic wheat lines was checked by Basta test strip, and the positive transgenic lines were further confirmed by PCR amplifying the *3* × *Flag-EsMYB90* sequence using the specific primers of EsMYB90-F/R (Supplementary Table 1). Thirty-seven independent T1 transgenic wheat lines overexpressing ectopically *EsMYB90* were obtained. Among these, the molecular identification of T1-4, T1-6, T1-7, T1-8, T1-9, and T1-30 transgenic wheat lines is shown in Supplementary Figure 1.

### Plant Materials and Salt Treatment

The wild-type (WT) wheat (*Triticum aestivum L.*) cv JW1 was provided by the Shandong Academy of Agricultural Sciences, and T3 homozygous transgenic lines were used to perform the salt stress treatment.

The seeds of wild type (WT) as well as EsMYB90 transgenic wheats were surfacely sterilized respectively with 70% ethanol and washed with distilled water, and these seeds were further soaked using 1% H_2_O_2_. The sterilized seeds were germinated on filter paper moistened with sterile distilled water in the dark for 2 days. The germinated wheat seedlings with coincident growth were selected and transplanted into a rectangle box (1 L) containing 1/2 Hoagland solution of 0 and 200 mM NaCl, respectively. Nine WT and 9 transgenic seedlings planted in 1/2 Hoagland solution for 10 days were the control group, while another 9 WT and 9 transgenic seedlings cultured in 1/2 Hoagland solution containing 200 mM NaCl for 10 days were the salt-treated groups. Subsequently, they were transferred to a growth chamber and grown at 22°C with 16 h light/18°C with 8 h dark. The control and salt-treated groups separately represented by three biological replicates were used to observe and photograph the phenotypes of wheat seedlings and survey the contents of chlorophyll, anthocyanin, and total flavonoid, as well as the root length, plant height, fresh weight, antioxidant enzyme activity, MDA content, and H_2_O_2_ accumulation.

The wheat seedlings were germinated on filter paper moistened with sterile distilled water in the dark at 22°C for 2 days; then were transplanted into 1/2 Hoagland solution without (control group) or with 200 mM NaCl (salt-treated group) for 24 h. For each treatment, the leaf sheaths of 9 plants were sampled and pooled to minimize the effect of transcriptomic unevenness among plants. The leaf sheath tissues of the control (CK) and salt-treated groups (NaCl) were respectively collected for RNA-seq and quantitative real-time (qRT)-PCR.

### Measurement of Anthocyanin, Total Flavonoid, and Chlorophyll

Anthocyanin and total flavonoid were extracted and quantified using the method described by Neff and Chory with minor modifications (Neff and Chory, 1998; Wang et al., 2016). The absorbance of the samples was determined at 530, 657, and 535 nm respectively using a spectrophotometer (UV-1800, Shimadzu), and the methanol with 1% HCl was used as a blank control. The anthocyanin content (mg/g FW) was calculated according to the formula of (A_530_ −0.25 × A_657_)/fresh weight (g), while the total flavonoid content (mg/g FW) was measured based on the equation (1/958 × A_535_ × 10,000 × V)/fresh weight (g), where the V represents the total volume of the extract (ml).

For the determination of chlorophyll content, the absorbance of samples was detected at 663 nm and 645 nm, where 80% acetone was used as the blank control. The chlorophyll content (mg/g FW) was calculated by the eq. (20.21 × A_645_ + 8.02 × A_663_)/fresh weight (g) (Şükran et al., 1998).

### Analysis of Growth, Antioxidant Enzymes Activity, Malondialdehyde, and H_2_O_2_ Accumulation in Transgenic Wheat Plants

To investigate the antioxidant capacity of transgenic wheat plants over-expressing *EsMYB90* and the important regulation role of *EsMYB90* gene plays in transgenic wheat, we detected the root length, plant height, and fresh weight to evaluate the salt tolerance of the wheat plants. Furthermore, the activity of glutathione transferase (GST) and POD antioxidant enzymes, the content of MDA, and the accumulation level of H_2_O_2_ were examined in the transgenic plants compared with the WT under the control and 200 mM NaCl treatment conditions.

The leaves, leaf sheaths, and roots of wheat plants were rapidly weighed (0.5 g), and ground in an ice-cold mortar supplemented with 8 mL 0.05 M Na_2_HPO_4_/NaH_2_PO_4_ buffer (pH 7.0). The homogenates were centrifuged at 4°C for 20 min, and the supernatants were collected to determine the POD activity and MDA content according to the method of Zhang and Kirkham (1994).

A glutathione transferase assay kit (Qiyi Biotechnology Co., Ltd., Shanghai) was used to determine GST activity. The 0.2 g wheat tissues to 1 ml reagent 1 (1:5 of weight/volume) were homogenized in an ice-bath, then the homogenate was centrifuged at 4°C for 10 min to take the supernatant. The sample tubes mixed with 100 μl sample supernatant, 900 μl reagent 2, and 100 μl reagent 3 were used to determine the absorbance of A3 (10 s) and A4 (310 s) at 340 nm. The blank tube mixed with 100 μl reagent 1, 900 μl reagent 2, and 100 μl reagent 3 was used to measure the absorbance of A1 (10 s) and A2 (310 s) at 340 nm. The absorbance was checked by using a spectrophotometer (UV-1800, Shimadzu). The GST enzyme activity (U/g FW) was calculated based on the formula as follows: 230 × [(A4−A3)–(A2−A1)] ÷W (fresh weight, g).

The 10-day salt treated wheat seedlings were soaked in a 1 mg/ml solution of 3, 3-diaminobenzidine (DAB)-HCl (PH 3.8), and vacuumed for 30 min to make the plants completely immersed in the DAB dye solution. Subsequently, they were incubated and stained at 25°C in the dark for about 12 h, then decolorized with 95% ethanol in a 60°C water bath for about 30 min, and the H_2_O_2_ accumulation level of the wheat seedlings was recorded by taking pictures (Ke et al., 2018).

In addition, an H_2_O_2_ content assay kit (Qiyi Biotechnology Co., Ltd., Shanghai) was employed to determine H_2_O_2_ content. The 0.1 g wheat root tissues were added to 1 ml reagent 1 (1:10 of weight/volume), and the mixture was homogenized in an ice-bath. Additionally, the homogenate was centrifuged at 4°C for 10 min before taking the supernatant. Subsequently, the total sample supernatant was added with 100 μl reagent 2 and 200 μl reagent 3, as the sample tube. The blank tube contains 1ml reagent 1, 100 μl reagent 2, and 200 μl reagent 3, while the standard tubes contain 100 μl reagent 2, and 200 μl reagent 3 added with 1 ml of 1 mmol/mL H_2_O_2_ standard solution. The mixtures of the blank (A0), sample (A1), and standard tubes (A2) were employed to determine the absorbance at 415 nm using the spectrophotometer (UV-1800, Shimadzu). The content of H_2_O_2_ was calculated according to the equation of [(A2-A0)/(A1-A0)]/W (fresh weight, g).

### Library Construction and Illumina Sequencing

Total RNA was extracted from the wheat leaf sheath tissues using RNAiso Reagent (Tiangen, China) according to the manufacturer’s instruction, and treated with DNase I to eliminate the DNA contamination. Sequencing libraries were generated using NEBNext^®^ Ultra™ RNA Library Prep Kit for Illumina^®^ (NEB, United States) according to the manufacturer’s instructions and the adaptors were added to attribute sequences to each sample. In the study, six cDNA libraries of wheat including the leaf sheaths from the wild-type (WT_NaCl1, WT_NaCl2, WT_NaCl3) and the transgenic plants (T_NaCl1, T_NaCl2, T_NaCl3) treated with 200 mM NaCl for 24 h were sequenced using the Illumina Novaseq 6,000 platform (Novogene company, Beijing), and 150 bp paired-end reads were generated.

### Mapping of RNA Sequencing Reads and Gene Expression Analysis

Raw reads were processed by removing the low-quality reads, adapter contamination, and the reads containing high content of unknown bases (N) to get clean reads using FastP software (Chen et al., 2018). After quality and adapter trimming, the clean reads were mapped to the reference genome of *Triticum aestivum*^1^ using the Hisat2 program based on no more than two mismatched bases in the alignment (Kim et al., 2019). The FPKM (Fragments Per Kilobase Million) of each transcript was calculated and normalized using featureCounts software, representing the gene expression level (Liao et al., 2014). DESeq2 software was employed to identify the differentially expressed genes (DEGs) by comparing the gene expression levels between the WT and transgenic lines (Love et al., 2014). The FoldChange ≥ 2 and padj (*q*-value) ≤ 0.05 were used as thresholds to screen the significant DEGs. The function of DEGs was annotated using the Nr database^2^.

### Gene Ontology and Kyoto Encyclopedia of Genes and Genomes Enrichment Analysis of Differentially Expressed Genes

Blast2GO program was used to annotate the Gene Ontology (GO) terms of DEGs against the GO database^3^. In the Kyoto Encyclopedia of Genes and Genomes (KEGG) enrichment analysis, the DEGs were mapped to specific biochemical pathways against the KEGG database^4^ (Kanehisa and Goto, 2000).

The GO and KEGG enrichment analysis of DEGs were implemented by the clusterProfiler R package (Yu et al., 2012), and the DEGs significantly enriched in GO terms and KEGG pathways were identified. The enrichment results of GO and KEGG were visualized by the ggplot2 R package.

### Quantitative Real-Time PCR

To validate the transcriptome results, qRT-PCR was performed by the LightCycler^®^ 96 system (Roche, Switzerland) to examine the expression of 15 selected genes including 3 *TaGST*, 4 *TaPOD* genes, and 8 other genes employing the same RNAs of the RNA-seq extracted from wheat leaf sheaths. Specific primers of the 15 genes were designed by Primer Premier 5.0 software, as shown in Supplementary Table 1, and the *Tatub4* (*tubulin beta-4*, *TraesCS5A02G416400*) was used as an internal control gene. The amplification program of the qPCR was as follows: 94°C for 10 min, followed by 40 cycles at 95°C for 15 s, 60°C for 10 s, and 72°C for 25 s. The expression of each gene was performed using three technical replicates (with three biological replicates), and the relative expression levels of genes were calculated according to the 2^–ΔΔ*CT*^ method (Livak and Schmittgen, 2001). Transcriptome data was verified by comparing the result of qRT-PCR (−ΔΔCT) with RNA-seq (log_2_FC), and Pearson’s correlation coefficient (PCC) was calculated using the cor.test function of the R base package.

### Dual Luciferase Assay

The online software PlantCARE^5^ was employed to predict conserved MYB-binding *cis*-element motifs located in the promoters of *TaANS2* and *TaDFR1* genes (named *pTaANS2* and *pTaDFR1*, respectively).

The regulation of EsMYB90 transcription factor (TF) on the down target genes was performed by dual luciferase assay. First, the promoters (−2000 to 0) of *TaANS2* (*TraesCS6D02G004300*) and *TaDFR1* (*TraesCS3B02G257900*) genes were cloned and recombined into the multiple cloning sites (MCS) of *pGreenII0800-LUC* as reporter vectors. The ORF of the *EsMYB90* gene was inserted into the *pCAMBIA3301H* vector to obtain *pCAMBIA3301H-p35S:EsMYB90* as the effector vector and the empty *pCAMBIA3301H* vector was used as a control. Primers for these constructs are listed in Supplementary Table 1. Then, these constructs were separately transferred into *Agrobacterium tumefaciens* GV3101. Furthermore, the GV3101 containing the reporter gene vector of *pGreenII0800-pTaANS2* (or *pTaDFR1*)*:LUC* was co-transfected with GV3101 of *pCAMBIA3301H-p35S:EsMYB90* (or *pCAMBIA3301H)* effector vector into the leaves of tobacco (*Nicotiana benthamiana*). After 2 − 3 days of the tobacco leaves being transfected, the enzyme activities of firefly luciferase (*pTaANS2*:*LUC* or *pTaDFR1*:*LUC*) and renilla luciferase (*p35S:REN*) were separately determined by the dual-luciferase reporter assay system (Meilunbio), in a highly sensitive plate chemiluminescence instrument (Centro LB960, Berthold Technologies, Wildbad, German). Finally, the relative luciferase activities (LUC/REN) from the experiment groups (EsMYB90) using *pCAMBIA3301H-p35S:EsMYB90* as the effector and the control group (CK) using *pCAMBIA3301H* as the effector, were calculated by the ratio of LUC to REN, respectively. The regulation efficiency of EsMYB90 TF on target genes was analyzed by the ratio of LUC/REN in the EsMYB90 group vs. that in the control group. Each assay was carried out using three independent experiments, and each independent experiment was conducted with three biological replicates. Error bars are the SE of the three biological replicates.

### Transcription Factor Analysis

The plant transcription factors were identified and classified according to their consensus sequences that were mainly summarized from plnTFDB and plantTFDB using the iTAK software (Pérez-Rodríguez et al., 2010; Jin et al., 2016; Zheng et al., 2016). The expression and classification of the transcription factor genes were visualized by TBtools software (Chen et al., 2020).

### Statistical Analysis

Statistical significance was performed using a Student’s *t*-test by the R base package, and the significant differences relative to the controls are indicated in *P ≤ 0.05, ^**^P ≤ 0.01, and ^***^P ≤ 0.001. The mean values and standard deviations (SDs) were calculated from three biological replicates.

## Results

### *EsMYB90* Gene Enhances the Flavonoid Content in the Leaf Sheath Tissues of Transgenic Wheat

To investigate the important role of the *EsMYB90* gene in improving the antioxidant capacity of transgenic wheat under salt stress, we generated the *Ubiquitin*:3 × *Flag-EsMYB90* (*Ubi:EsMYB90*) construct to ectopically express *EsMYB90* in wheat plants, and 37 independent T1 transgenic lines were obtained by *Agrobacterium*-mediated genetic transformation.

**FIGURE 1:**
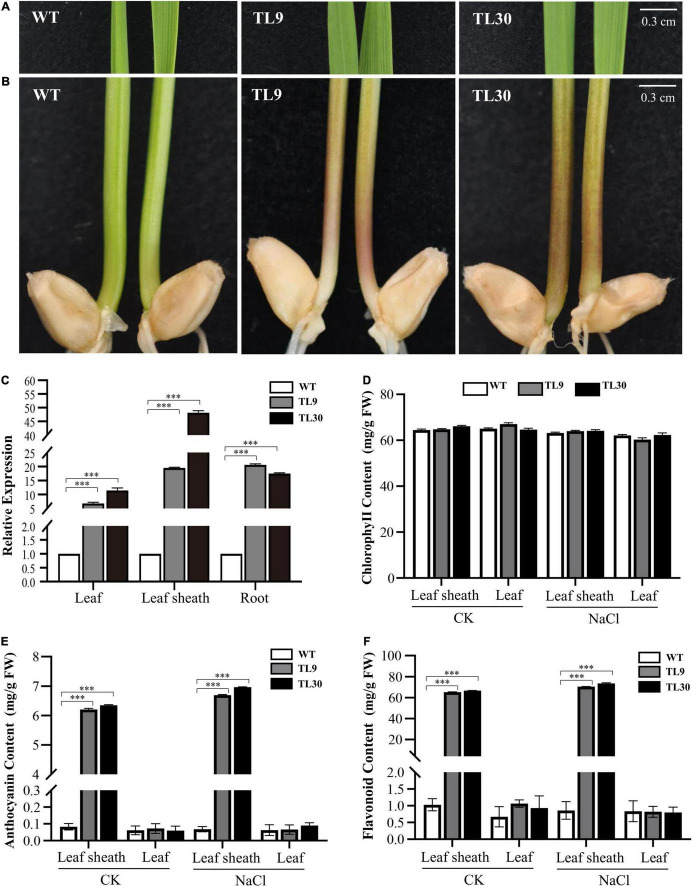
Expression of *EsMYB90* gene enhanced contents of anthocyanin and total flavonoid in leaf sheath tissues of transgenic wheat plants. The color phenotype of leaves **(A)** and leaf sheaths **(B)** in TL9 and TL30 transgenic lines compared with wild type (WT), from 10-day-old wheat plants. Bars:0.3 cm. **(C)** Expression analysis of *EsMYB90* in leaf, leaf sheath, and root tissues of transgenic lines compared to WT by quantitative real-time (qRT)-PCR. *Y*-axis indicates the relative expression of the *EsMYB90* gene calculated by 2^–ΔΔCT^. *Tatub4* (*tubulin beta-4*, *TraesCS5A02G416400*) was used as an internal control gene. Contents of chlorophyll **(D)**, anthocyanin **(E)**, and total flavonoid **(F)** in leaf and leaf sheath tissues of transgenic lines compared with WT. Student’s *t-*test values are indicated as ^***^P < 0.001 and vertical bars indicate the SEs of three biological replicates.

The T3 homozygous transgenic wheat lines were used to determine the expression level of the *EsMYB90* gene by qRT-PCR. The result showed that TL9 and TL30 transgenic lines possessed a higher expression level of the *EsMYB90* gene. The expression of *EsMYB90* in the TL30 lines was increased by 11.5, 48.2, and 17.5 times in the leaves, leaf sheaths, and roots, respectively, compared with the WT (Figure 1C and Supplementary Table 2). Furthermore, we found that in 10-day-old wheat seedlings, the leaf sheaths of *EsMYB90* transgenic lines TL9 and TL30 lines produced an obvious purple-red phenotype, whereas the leaf sheath tissues of the wild-type were green (Figure 1B). However, the leaf tissues between the transgenic lines and wild-type plants were shown to be almost consistently green, which is in agreement with the non-obvious changed level of chlorophyll in transgenic wheat compared with the WT (Figures 1A,D).

Our results from examining the anthocyanin and total flavonoid production found that the contents of anthocyanin and total flavonoid, respectively, in two *Ubi:EsMYB90* wheat lines were all significantly increased in leaf sheaths compared with the WT. Compared with the WT, the anthocyanin contents in leaves and leaf sheaths of the L30 line were separately elevated by 1.0 and 77 times (Figure 1E and Supplementary Table 3), while the total flavonoid contents in the leaves and leaf sheaths of the L30 line were increased 1.4 and 64.8 times, respectively (Figure 1F and Supplementary Table 3). However, after the salt treatment of 200 mM NaCl for 10 days, the transgenic wheat lines showed almost unaffected contents of chlorophyll, anthocyanin, and total flavonoid in contrast to the normal growth condition (CK) (Figures 1A,B,D–F).

In summary, these results indicate that the leaf sheath tissues exhibited higher anthocyanin accumulation as well as the total flavonoid content, and the expression level of the *EsMYB90* gene was consistent with the enhanced contents of anthocyanin and total flavonoid in transgenic wheat plants.

### Ectopic Expression of the *EsMYB90* Gene Promotes Growth in Transgenic Wheat Subjected to Salt Stress

To investigate the functions of the *EsMYB90* gene in the transgenic wheats responding to salt stress, the TL9, TL30 transgenic lines, and WT wheat plants were planted in 1/2 Hoagland solution containing 0 and 200 mM NaCl, respectively, to survey and record their growth phenotype.

**FIGURE 2:**
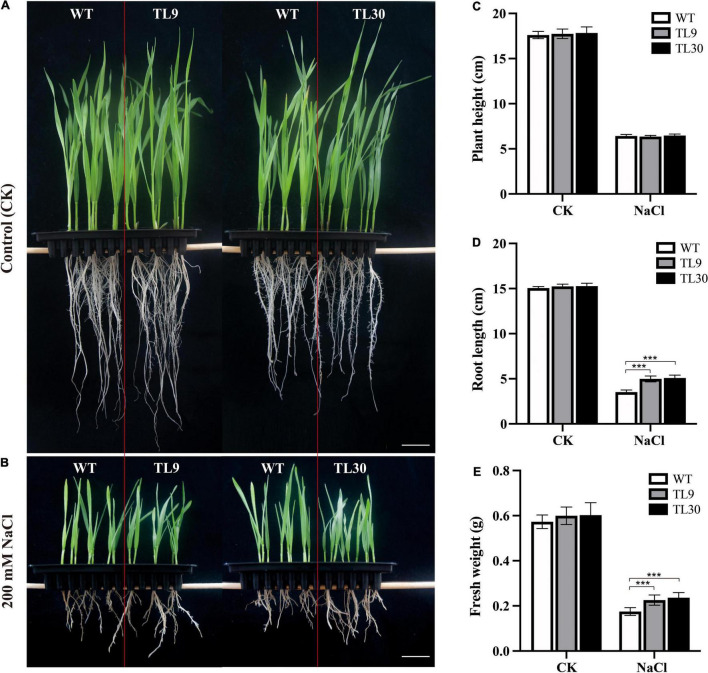
Effect of *EsMYB90* overexpression on the salt tolerance of the transgenic wheat. Two-day-old seedlings germinated on wet filter papers were transferred into 1/2 Hoagland solution, as a control group **(A)**, and transferred into 1/2 Hoagland solution containing 200 mM NaCl, as the treatment group **(B)**. The phenotypes of the wheat plants (TL9, TL30, WT) under the control (CK) and NaCl treatment (200 mM) were surveyed and photographed. Bar: 2 cm. Comparative analysis of root length **(C)**, plant height **(D)**, and fresh weight **(E)** among transgenic TL9, TL30, and WT line under 200 mM NaCl treatment and the normal growth condition. WT, wild type; TL9 and TL30, transgenic wheat lines. Mean values and deviations were calculated from three independent biological experiments. ***indicates *p* ≤ 0.001.

The result indicated that under 200 mM NaCl stress, the *EsMYB90* transgenic lines (TL9, TL30) were observed to have a significant increase (*P* ≤ 0.001) in root length and fresh weight when compared with the WT wheat, whereas, there was no obvious difference in the plant height (Figures 2B–E). No other difference in phenotypes between wild-type and ectopic *EsMYB90* overexpressing plants except for a deepening purplish-red phenotype in the leaf sheath tissues of transgenic lines were observed when grown under normal, non-salt-stressed conditions (Figures 2A,C–E). The result demonstrated the *EsMYB90* gene strikingly increased the root length and fresh weight of transgenic wheat subjected to salt stress, indicating that the gene is possibly involved in the regulation of salt tolerance.

### *EsMYB90* Gene Enhances the Antioxidant Capacity of Transgenic Wheat

Previous studies documented that the *EsMYB90* gene could significantly increase the flavonoid content of transgenic wheat plants (Qi et al., 2021). In the study, we further detected the activities of GST and POD antioxidant enzymes, and the oxidative damage extent in transgenic wheat lines compared with the WT under salt stress.

**FIGURE 3:**
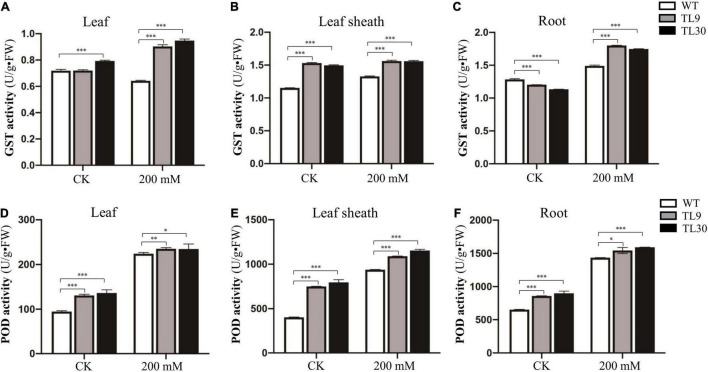
Antioxidant enzyme activity analysis in the different tissues of transgenic wheat lines and WT plants under salt stress and normal growth conditions. The GST activity in leaves **(A)**, leaf sheaths **(B)**, and roots **(C)**, and the POD activity in leaves **(D)**, leaf sheaths **(E)**, and roots **(F)** are shown in the Figure. TL9 and TL30, the transgenic wheat lines; WT, wild type. Mean values and deviations were calculated from three independent biological experiments. *p ≤ 0.05, ^**^p ≤ 0.01, and ^***^p ≤ 0.001 indicate significance in transgenic wheat lines relative to WT.

**FIGURE 4:**
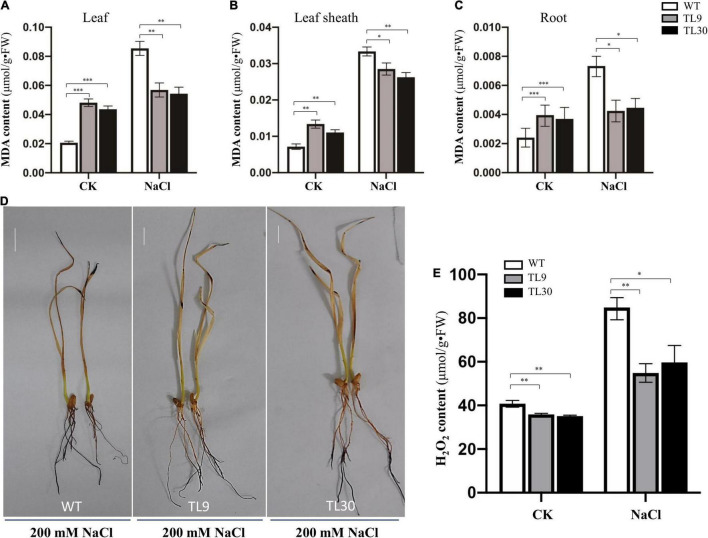
Analysis of oxidative damage extent in transgenic wheat lines compared to WT plants under salt stress conditions. MDA contents in leaves **(A)**, leaf sheaths **(B)**, and roots **(C)**. Mean values and deviations were calculated from three independent biological experiments. *p ≤ 0.05, ^**^p ≤ 0.01, and ^***^p ≤ 0.001 indicate significance in transgenic wheat lines relative to WT. **(D)** H_2_O_2_ accumulation level detected by DAB histochemical staining. **(E)** H_2_O_2_ content was determined in the roots of the 10-day-old wheat seedlings using an H_2_O_2_ content assay kit. TL9 and TL30, the transgenic wheat lines; WT, wild type.

The result demonstrated that, after 200 mM NaCl treatment for 10 days, the GST and POD activities were all significantly higher in the leaves, leaf sheaths, and roots of TL9 and TL30 transgenic lines than that in the WT (Figures 3A–F and Supplementary Table 4). In normal growth conditions, the POD activity in the leaves, leaf sheaths, and roots, as well as the GST activities in the sheaths were also markedly higher in the TL9 and TL30 transgenic lines compared with the wild type (Figures 3A–F and Supplementary Table 4). MDA is the product of membrane lipid peroxidation, and its excessive accumulation results in the damage of cells and membrane structure (Dhindsa et al., 1981). Generally, the decrease of MDA content in plant tissues indicates the reduced oxidative damage degree caused by the stress stimuli. Our research found that the MDA contents in leaves, leaf sheaths, and roots of transgenic wheat TL9 and TL30 lines were significantly lower than those in the wild-type plants after 200 mM NaCl treatment for 10 days (Figures 4A–C and Supplementary Table 4).

Additionally, when the plants were treated with 200 mM NaCl for 10 days, the H_2_O_2_ accumulation level of wheat tissues was detected by the DAB (3,3′-diaminobenzidine) histochemical staining, and the result exhibited that there existed a deeper brown-dark in almost the entire root tissues and leave tips in the WT wheat plants, while there was barely brown-dark in the upper half of roots in transgenic wheat (Figure 4D), which indicated that there was much more H_2_O_2_ accumulation in the WT plants than that in the TL9 or TL30 transgenic lines (Figure 4D). Meanwhile, the H_2_O_2_ content in the root tissue of wheat seedlings was determined using an H_2_O_2_ content assay kit, and the results documented that, under both the control and 200 mM NaCl treatment conditions, the H_2_O_2_ contents were all markedly lower in the *EsMYB90* transgenic lines than that in the WT; this result was consistent with the lower H2O2 level in the *EsMYB90* transgenic wheat lines by DAB staining method (Figures 4D,E).

Collectively, these results demonstrated that the *EsMYB90* gene remarkably enhanced the antioxidant enzyme activities of GST and POD, and reduced the injury extent of salt stress to membrane lipid in transgenic wheat subjected to 200 mM NaCl treatment for 10 days, suggesting a significantly improved antioxidant capacity of transgenic wheat plants under salt stress.

### RNA Sequencing Analysis in the Leaf Sheaths of Transgenic Wheat Upon Salt Shock

**FIGURE 5:**
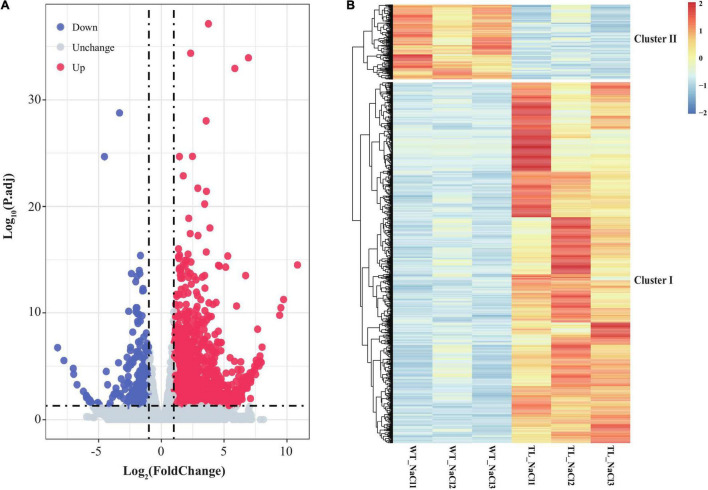
Volcano plot and expression patterns of differential transcripts in leaf sheaths of *EsMYB90* transgenic tobacco relative to wild type. **(A)** Volcano plot exhibiting the differential expression level of transcripts detected in the RNA-seq. The abscissa represents the logarithm of fold change (TL/WT) of transcripts (log2FC) under 200 mM NaCl; the ordinate indicates the –log_10_ (p.adj). Red dots represent the upregulated differential transcripts; Blue dots represent the downregulated differential transcripts; Light gray dots represent the transcripts that were detected with no significant difference. **(B)** The heat map displays the distinguished expression patterns of the differential expressed genes (DEGs). The transcript’s abundance calculated as FPKM is shown in the color legend, where red indicates the transcripts with a higher level, and blue indicates transcripts with a lower level.

To clarify the function and possible regulation mechanism of the *EsMYB90* gene in the response of transgenic wheat to salt stress, we conducted the RNA-seq analysis of leaf sheaths in the transgenic TL30 line and wild-type wheat upon 200 mM NaCl for 24 h using the Illumina Novaseq6000 platform. First, the clean data, which represented more than 99% of the raw data, were obtained after removing the adapter, poly-N, and low-quality reads. The total number of clean reads from the wild-type (WT_NaCl1, WT_NaCl2, WT_NaCl3) and the transgenic plants (T_NaCl1, T_NaCl2, T_NaCl3) was 473,304,684, with an average of ∼79 million reads per library (Table 1). Approximately 94.85% of clean reads from the leaf sheath tissues could be mapped to the reference genome, among which 90.4% of clean reads were uniquely mapped to the reference genome (Table 1). Furthermore, a total of 2,175 DEGs, including 1,802 upregulated and 373 downregulated genes, according to the criterion of log_2_FC ≥ 1 (or ≤ −1) and Padj ≤ 0.05, were identified in transgenic wheat compared with the WT under 200 mM NaCl (Figure 5A and Supplementary Table 5).

**TABLE 1:** Summary of reads mapping to reference genome.

Samples	Total clean reads	Total mapped reads	Unique match	Multi-position match	Unmapped reads
WT_NaCl1	79,882,976	75,288,011 (94.25%)	71,881,369 (89.98%)	3,406,642 (4.26%)	4,594,965 (5.75%)
WT_NaCl2	79,470,912	75,346,724 (94.81%)	71,829,139 (90.38%)	3,517,585 (4.43%)	4,124,188 (5.19%)
WT_NaCl3	79,995,596	76,140,776 (95.18%)	72,326,527 (90.41%)	3,814,249 (4.77%)	3,854,820 (4.82%)
T_NaCl1	77,928,978	73,710,398 (94.59%)	70,341,196 (90.26%)	3,369,202 (4.32%)	4,218,580 (5.41%)
T_NaCl2	76,317,154	72,674,247 (95.23%)	69,259,462 (90.75%)	3,414,785 (4.47%)	3,642,907 (4.77%)
T_NaCl3	79,709,068	75,735,333 (95.01%)	72,263,982 (90.66%)	3,471,351 (4.36%)	3,973,735 (4.99%)

To acquire an overview of the salt-response genes in *EsMYB90* transgenic wheat, we illuminate the differential expression patterns of these DEGs by hierarchical clustering analysis using heatmap, and the two major characteristics of cluster I and cluster II were distinguished (Figure 5B and Supplementary Table 5). The result showed that there were more upregulated genes (cluster I) and relatively few downregulated genes (cluster II) in the *EsMYB90* transgenic line compared with the WT upon salt stress (Figure 5B and Supplementary Table 5). In comparison with WT wheats, all DEGs such as cytochrome P450 genes *F3*′*5*′*H* (*TraesCS6B02G405900*, *TraesCS6B02G406400*) and *F3*′*H* (*TraesCS7D02G404900*, *TraesCS5B02G209100*) associated with flavonoids biosynthesis in cluster I were significantly up-regulated in *EsMYB90* transgenic line after NaCl treatment (Figure 5B and Supplementary Table 5). On the contrary, the DEGs such as the late blight resistance protein coding genes *R1B-14* (*TraesCS2A02G565800*) and *R1B-12* (*TraesCS2A02G564200*) from cluster II were all remarkably downregulated in the transgenic wheat upon NaCl stress (Figure 5B and Supplementary Table 5).

**FIGURE 6:**
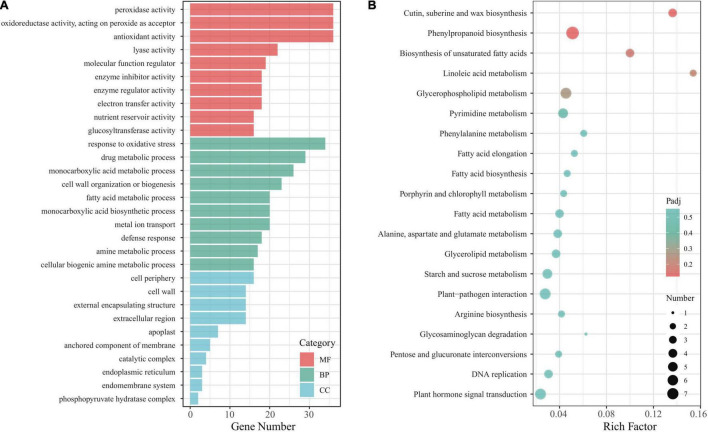
Function analysis of DEGs in leaf sheaths of *EsMYB90* transgenic wheat compared with wild type subjected to 200 mM NaCl treatment. **(A)** Histogram analysis of GO terms. MF, molecular function; BP, biological process; CC, cellular component. **(B)** Analysis of top 20 KEGG pathways. The rich factor is the ratio of the DEGs numbers to the total of annotated genes in a certain pathway. In a specific KEGG pathway, the color of dots represents the significance of DEGs, and the size of dots indicates the number of DEGs. Padj indicates the significance of the enriched DEGs, and the smaller padj value indicates higher significance.

To understand in-depth the function of the *Eutrema EsMYB90* gene and its influence on these DEGs in the leaf sheaths of transgenic wheat under salt stress, the GO terms of the DEGs were annotated and analyzed. The result showed that 740 unique DEGs were annotated to 678 GO terms. The top 10 GO terms in the biological process (BP), cellular component (CC), and molecular function (MF) were selected for visualization in a histogram (Figure 6A). The ‘POD activity,’ ‘oxidoreductase activity,’ and ‘antioxidant activity’ were the three main GO terms in the molecular function, and the ‘response to oxidative stress’ was the most markedly enriched GO term in the biological process. In cell component classification, the main terms include ‘cell periphery,’ ‘cell wall,’ and ‘external encapsulating structure’ (Figure 6A and Supplementary Table 6). Furthermore, the enrichment analysis of KEGG pathways was performed, and 72 DEGs were assigned to 53 KEGG pathways. Among the represented top 20 KEGG pathways, the ‘cutin, suberine, and wax biosynthesis’ (ko00073), ‘phenylpropanoid biosynthesis’ (ko00940), and ‘biosynthesis of unsaturated fatty acids’ (ko01040) were the most significantly enriched pathways (Figure 6B and Supplementary Table 7).

Altogether, the GO and KEGG analysis indicated that the DEGs involved in enhancing antioxidative capacity, and biosynthesis of cutin, wax, and unsaturated fatty acids could play a critical role in the response of *EsMYB90* transgenic wheat to salt stress.

### *EsMYB90* Promotes the Expression of Genes Involved in Antioxidant and Cell Wall Plasticity in Transgenic Wheat Upon Salt Stress

Glutathione S-transferase could scavenge external toxins and endogenous toxic metabolites in plants (Islam et al., 2018). Additionally, GSTs as the ligandins are involved in anthocyanin transport, indicating they could play a key role in increasing the antioxidant capacity of plants (Conn et al., 2008). The POD could scavenge ROS produced in a cell responding to salt stress and plays an important role in reducing the toxicity of ROS to organelles (Hassan and Ali, 2014).

**FIGURE 7:**
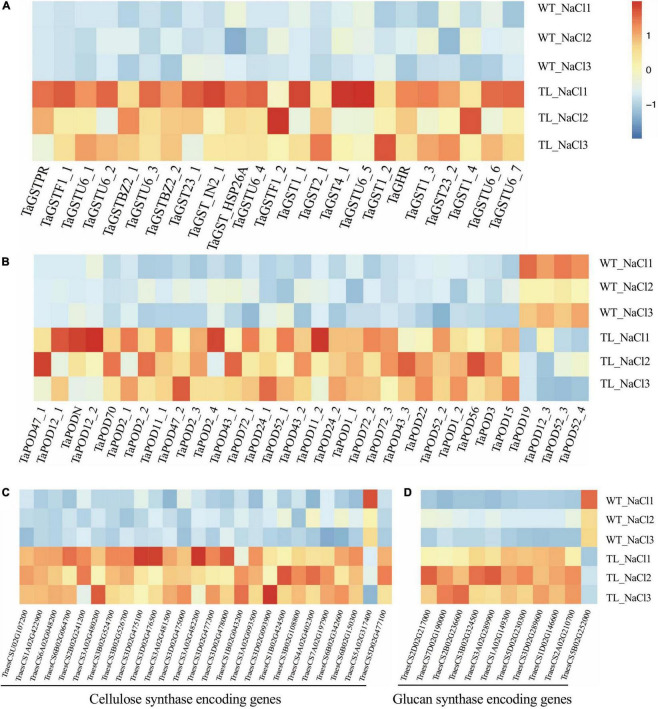
Expression analysis of antioxidant enzyme encoding genes in the leaf sheath tissue of transgenic wheat upon salt stress in RNA-seq using a heat map. Expression analysis of glutathione transferase coding gene *GST* **(A)**, peroxidase (POD) coding gene *POD* **(B)**, cellulose synthase coding genes **(C)**, and glucan synthase coding genes **(D)**. The color legend indicates the expression level of DEGs (FPKM), where the redder the color represents the higher gene expression level, and the darker blue color represents the lower gene expression level.

In this study, we first identified the GST and POD encoding genes at the genome level by annotating the DEGs of RNA-seq from the leaf sheaths of transgenic wheat compared with the WT upon salt stress using the Nr database. Furthermore, the heatmap analysis indicated that the transcript level of 23 *GST* genes and 28 *POD* genes identified were remarkably enhanced in transgenic wheat upon salt stress. Among which, the expression of GST coding genes (*TraesCS3D02G486800*, *TraesCS7D02G514500*, *TraesCS2B02G523700*, *TraesCS1A02G186600*, and *TraesCS7D02G348500*) were strikingly upregulated by 73.6, 54, 27.3, 19.1, and 10 times, respectively (Figure 7A and Supplementary Table 8). The transcript abundance of POD coding genes (*TraesCSU02G137300*, *TraesCS2B02G614100*, *TraesCS1B02G115800*, *TraesCS2A02G573500*, and *TraesCS7D02G370400*) were separately elevated by 82.3, 62.5, 16.1, 7.9, and 7.6 times (Figure 7B and Supplementary Table 8).

The plants could alleviate the damage of water stress on cells and improve their tolerance to salt stress *via* regulating the relaxation and increasing the rigidity of the cell wall (Byrt et al., 2018). In monocots, the (1, 3-1, 4)-β-endoglucanase and β-glucosidases were reported to be associated with the lignin biosynthesis that affects the rigidity of the cell wall (Zhu et al., 2007). The adaptation of plants to high concentrations of NaCl and osmotic stress requires the changes in cell wall extensibility that are involved in the function of endo-β-1, 4-glucanases (Boucaud et al., 1987; Nicol et al., 1998). Our transcriptome analysis illustrated that in transgenic *EsMYB90* wheat compared with the WT upon salt shock (200 mM NaCl, 24h) the expression of the cellulose synthase encoding genes *CESA* (*TraesCS2D02G217000* and *TraesCS7D02G190000*) was significantly upregulated (Figure 7C and Supplementary Table 8). Meanwhile, the expression level of glucan metabolism related genes such as glucan endo-1,3-β-glucosidase coding genes *GEBG* (*TraesCS3A02G480200*, *TraesCS3A02G482200*, *TraesCS3D02G477300*, and *TraesCS3B02G524700*) were strikingly enhanced by 47.2, 32.3, 18.9, and 16.9 times, respectively, and the transcripts of endoglucanase coding genes *EG* (*TraesCS6B02G150300*, *TraesCS7A02G197900*, and *TraesCS6B02G342600*) were also markedly elevated (Figure 7D and Supplementary Table 8).

Taken together, the RNA-seq results demonstrated that *EsMYB90* gene strongly increased the expression of antioxidant related genes (*GST*, *POD*) as well as the cellulose and glucan metabolism related genes in leaf sheaths of transgenic wheat under salt stress. This suggested that *EsMYB90* played a key regulatory role in enhancing the oxidative capacity and cell wall plasticity in the transgenic wheat in response to salt stress *via* increasing the expression level of the genes associated with antioxidant and cell wall extensibility.

### Validation of RNA-Seq Result and Expression Analysis of Stress-Related Genes Using Quantitative Real-Time-PCR

**FIGURE 8:**
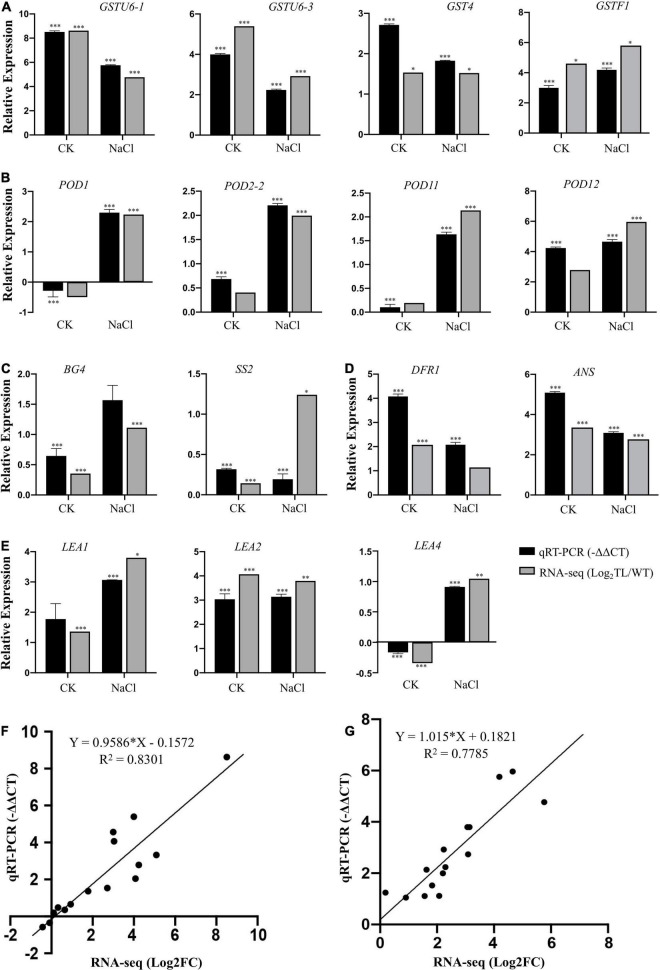
Validation of RNA-seq result using qRT-PCR. Expression verification of 15 genes including **(A)** *GST* genes, **(B)** *POD* genes, **(C)** Sucrose and glucans metabolism related genes, **(D)** Anthocyanin biosynthesis related genes, **(E)** LEA protein encoding genes, in RNA-seq by qRT-PCR. Black bars indicate −ΔΔCT of DEGs in qRT-PCR, and gray bars show log_2_FC (TL/WT) of DEGs in RNA-seq. The *Tatub4* (*tubulin beta-4*, *TraesCS5A02G416400*) was used as an internal control gene. Each set of data was obtained from three biological replications, and the vertical bar represents the standard error of 3 biological replications. *p ≤ 0.05, ^**^p ≤ 0.01, and ^***^p ≤ 0.001 indicate the significance of gene expression in transgenic wheat plants compared with the WT under salt stress. Correlation analysis of results between RNA-seq [X-axis:log_2_ FC (TL/WT)] and qRT-PCR (Y-axis: –ΔΔCT) in the normal condition **(F)** and NaCl treatment condition **(G)**.

To confirm the reliability of the RNA-seq data and further validate the regulatory effect of the *EsMYB90* gene under salt stress, we analyzed the relative expression of 15 DEGs in the leaf sheaths of transgenic wheat compared with WT by qRT-PCR. These DEGs are mainly related to GST and POD enzymes, carbohydrate metabolism, anthocyanin biosynthesis, and LEA proteins (Supplementary Table 9). The quantitative PCR results showed that the expression of glutathione transferase coding genes *GSTU6-1* (*TraesCS2B02G523700*),*GSTU6-3* (*TraesCS1A02G186700*), and *GST4* (*TraesCS5A02G024100*) in transgenic wheat were significantly higher than that in the WT with 54.2, 4.7, and 3.5 times under salt stress, and 364.5, 16, and 6.6 times under normal growth conditions, respectively (Figure 8A and Supplementary Table 9). Under salt stress, the expression levels of POD coding genes *POD1* (*TraesCS2B02G124300*),*POD2-2* (*TraesCS7D02G461200*),*POD11* (*TraesCS7A02G211200*), and *POD12* (*TraesCS2B02G614100*) in transgenic wheat were significantly higher than that in the WT plant with 4.9, 4.6, 3.1, and 25.2 times, respectively (Figure 8B and Supplementary Table 8). Particularly, it can be seen that under salt stress and normal growth conditions, the expression levels of antioxidant enzyme coding genes *GSTU6-1* and *POD12* in *EsMYB90* transgenic wheat were strikingly increased more than 18-fold than that in the WT (Figures 8A,B and Supplementary Table 9). The result indicated that *EsMYB90* could strongly improve the antioxidant capacity of transgenic wheat by enhancing the expression of antioxidant and toxins scavenging genes.

In addition, the expression levels of β-1,3-glucanase 4 (*BG4*, *TraesCS6B02G064700*) and sucrose synthase 2 (*SS2*, *TraesCS2B02G194200*) encoding genes in transgenic wheat were significantly higher than that in the wild type both in the NaCl treatment and normal growth conditions (Figure 8C and Supplementary Table 9). The transcript abundance of *DFR* encoding dihydroflavonol 4-reductase and *ANS* encoding anthocyanidin synthase, and LEA protein coding genes *LEA1* (*TraesCS3B02G408500*) and *LEA2* (*TraesCS2D02G561400*) were upregulated by 4.2, 8.5, 8.4, and 8.8 times in transgenic wheat compared with the wild type under salt stress, respectively (Figures 8D,E and Supplementary Table 9).

The relative expression levels (log_2_NaCl/CK) of 15 genes in RNA-seq were validated using RT-PCR (-ΔΔCT), and the results demonstrated that there was good consistency in the expression levels of these genes analyzed in the leaf sheaths of wheat in the salt treatment (*R*^2^ = 0.7785) and control (*R*^2^ = 0.8301) conditions between using qRT-PCR and RNA-seq, indicating that the results of the RNA-seq were trustworthy (Figures 8F,G).

### EsMYB90 Enhanced Flavonoid Content *via* Activating the Transcription of Flavonoid Biosynthesis Genes

**FIGURE 9:**
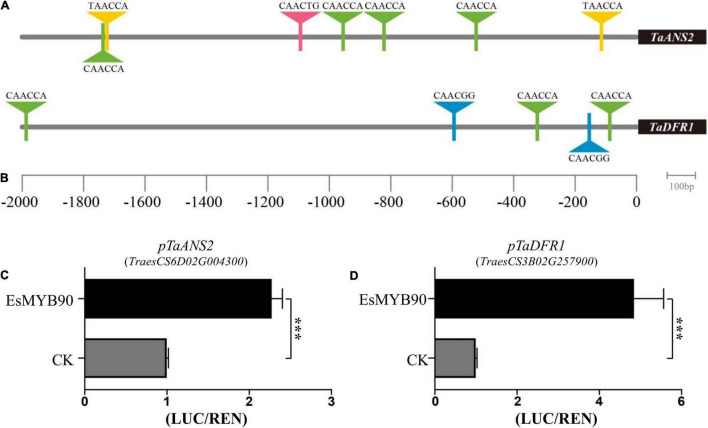
EsMYB90 activates the transcription of flavonoid biosynthesis genes in transgenic wheat. **(A)** MYB-binding motifs analysis in *TaANS2* promoter region. **(B)** MYB-binding motifs analysis in *TaDFR1* promoter region. **(A,B)** The triangle indicates the predicted MYB recognized *cis*-elements. **(C)** Relative luciferase activity (LUC/REN) of *TaANS2* promoter (*pTaANS2*) by EsMYB90. **(D)** Relative luciferase activity (LUC/REN) of *TaDFR1* promoter (*pTaDFR1*) by EsMYB90. The values are means ± SD of three biological replicates. Asterisks indicate significant difference: ***p ≤ 0.001.

Dihydroflavonol 4-reductase (DFR) and anthocyanidin synthase (ANS) have been considered the key enzymes of anthocyanin biosynthesis (Holton and Cornish, 1995). In order to clarify the regulatory mechanism of EsMYB90 transcription factor (TF) on the biosynthesis of anthocyanin, we first analyzed the MYB-binding motifs in the promoters of *TaANS2* (*pTaANS2*) and *TaDFR1* (*pTaDFR1*) genes in transgenic wheat using PlantCARE and visualized by Tbtools software. The results indicated that seven MYB transcript factor binding elements exist in *pTaANS2* and five in *pTaDFR1* (Figures 9A,B and Supplementary Table 10).

Furthermore, the GV3101 containing *pCAMBIA3301H-p35S:EsMYB90* (or *pCAMBIA3301H* as control) as an effector, and the GV3101 containing *pGreenII0800-pTaANS2:LUC* or *pGreenII0800-pTaDFR1:LUC* as the reporter were co-transfected into tobacco leaves. And the dual luciferase assay revealed that the relative activity ratio of pTaANS2:LUC firefly luciferase to renilla luciferase (p35S:REN) was upregulated about 2.3 times by EsMYB90 TF (Figure 9C and Supplementary Table 11), while the ratio of pTaDFR1:LUC firefly luciferase to renilla luciferase (p35S:REN) was increased 4.9 times by EsMYB90 TF (Figure 9D and Supplementary Table 11).

This study demonstrated that the EsMYB90 transcription factor operating as a positive transcriptional regulator may directly bind to the MYB-binding elements of *pTaANS2* and *pTaDFR1* to activate the transcription of *TaANS2* and *TaDFR1* genes in the *EsMYB90* transgenic wheat that will lead to the biosynthesis of anthocyanidin and other flavonoids. Additionally, this result is in agreement with the enhanced level of anthocyanin and total flavonoids in leaf sheath tissues of transgenic wheat (Figures 1B,E,F).

## Discussion

### Function Analysis of EsMYB90 Protein by Phylogenetic Comparison With the Other R2R3-MYB

MYB proteins are a superfamily of transcription factors that play important regulatory roles in various development processes and defense responses in plants (Yanhui et al., 2006). The R2R3-MYB family containing the largest number of MYB genes was reported to be involved in different stress responses (Abe et al., 2003; Nagaoka and Takano, 2003).

**FIGURE 10:**
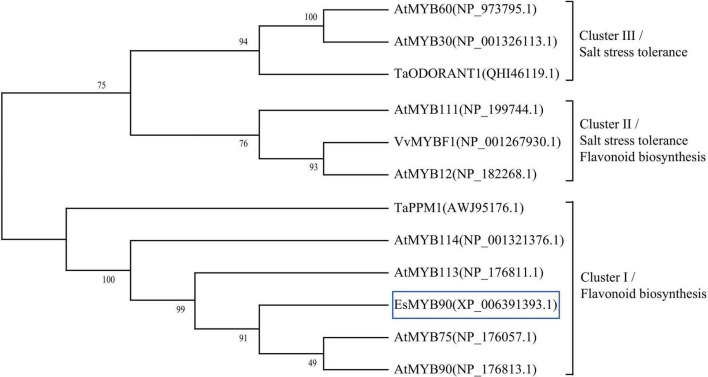
Phylogenetic analysis of EsMYB90 and the other 11 MYB proteins in plants. EsMYB90 is highlighted with a blue box. The MYB protein sequences were downloaded from the GenBank database with accession numbers shown in the diagram.

To further explore the potential regulatory function of *Eutrema* EsMYB90 in the stress tolerance of plants, we performed a phylogenetic analysis of EsMYB90 with other 11 MYB proteins possessing various functions. The results showed that *Eutrema* EsMYB90 protein had a higher phylogenetic relationship with *Arabidopsis* AtMYB75, AtMYB90, AtMYB113, AtMYB114, and wheat TaPPM1 proteins, functioning mainly in flavonoids biosynthesis (Figure 10). AtMYB12, AtMYB111, and VvMYBF1 proteins cluster in another evolutionary branch (Figure 10), and their function are not only involved in flavonoid biosynthesis but also in abiotic stress tolerance (Li et al., 2019; Wang et al., 2020, 2021). Meanwhile, the functions of AtMYB30, AtMYB60, and TaODORANT1 in cluster III are mainly associated with the regulation of salt tolerance in plants (Wei et al., 2017; Wang et al., 2021; Figure 10). Hence, it is suggested that EsMYB90 TF plays an important role in the enhanced growth and antioxidant capacity of transgenic wheat, which could be partially attributed to the increased flavonoids biosynthesis.

### Identification of Transcription Factor Genes in *EsMYB90* Transgenic Wheat Under Salt Shock

Transcription factors (TFs) are crucial regulators of gene expression, and function as molecular switches for the conversion of stress signal perception to stress-responsive gene expression (Zhao et al., 2020). In WRKY, bHLH, MYB, NAC, AP2/ERF-ERF, and bZIP families, some members have been reported to be involved in the enhanced salt stress tolerance of plants (Kiełbowicz-Matuk, 2012; Wang et al., 2016).

**FIGURE 11:**
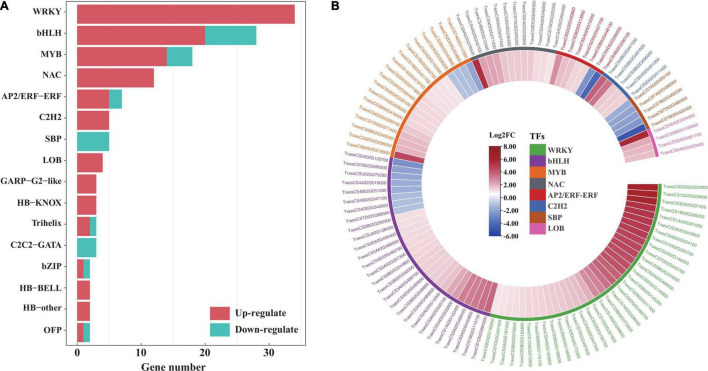
Analysis of transcription factors in the leaf sheaths of *EsMYB90* transgenic wheat compared with wild type upon salt shock. **(A)** Number distribution of the up/downregulated TF genes in different TF families. The red color bar indicates the upregulated and the blue color bar indicates the downregulated TF genes. **(B)** Expression analysis of the differential expressed TF genes in top 8 TF families using the heatmap. The differential transcript levels of TF genes calculated as Log_2_ (TL/WT) are shown in the color legend, where red represents upregulated and blue represents the downregulated genes. The gene ID of the different TF families was shown in different colors.

To provide insight into the TFs responding to salt stress in *EsMYB90* transgenic wheat compared to the wild type, the number, and expression of genes in various TF families annotated were displayed with a bar plot and heatmap. The result showed that 143 significant DEGs were annotated to 26 TF families including WRKY, bHLH, MYB, NAC, AP2/ERF-ERF, and C2H2 in transgenic wheat when compared with WT (Figure 11A and Supplementary Table 12). Among these, the expression of 114 TF genes increased more than two-fold upon salt stress. These upregulated TF genes mainly include WRKY (34), bHLH (20), MYB (14), NAC (12), AP2/ERF-ERF (5), and C2H2 (5) (Figure 11 and Supplementary Table 12).

The R2R3-MYB factors act as heterodimers with bHLH (basic helix-loop-helix) transcription factors and allow a high degree of plasticity in controlling their effect on ectopic expression (Dou et al., 2017). In our study, EsMYB90 significantly upregulated the expressions of 20 *TabHLHs* (*TraesCS1D02G094200*, *TraesCS1B02G113100*, *TraesCS3A02G440600*, and *TraesCS3A02G489400*) in transgenic wheat plants (Figure 10 and Supplementary Table 10), which suggested that the formation of a heterodimer between *EsMYB90* and different *bHLH* genes was possibly an important mechanism for enhancing antioxidative capacity under salt stress.

### Regulating Mechanism of EsMYB90 in the Response of Salt Stress in Transgenic Wheat

The exposure of plants to salinity is known to induce oxidative stress that produces ROS in plants (Hajiboland and Joudmand, 2009). Plants employ different mechanisms to reduce the oxidative damage caused by ROS and provide necessary protection against innumerable toxic agents in environmental stresses, which is critically important for plants to maintain their proper development and growth (Abdel Latef, 2010). The induction of ROS-scavenging enzymes such as POD, SOD, and APX is the most common mechanism for detoxifying the ROS produced during stress response (Latef and Chaoxing, 2011; Hassan and Ali, 2014). GSTs are a ubiquitous superfamily of multifunctional enzymes involved in the detoxification of xenobiotics and stress metabolism (Mohsenzadeh et al., 2011). For instance, the overexpression of *GsGST* from *Glycine soja* enhances salt tolerance in transgenic tobacco (Ji et al., 2010), and the ectopic expression of soybean *GmGSTL1* in *Arabidopsis* alleviates the symptoms of salt stress (Chan and Lam, 2014). Moreover, the function of GSTs is also indelibly involved in anthocyanin transport, such as the characterization of glutathione S-transferases (GSTs) from pigmented *Vitis vinifera* cell suspension cultures that indicated *VvGST1* and *VvGST4* could be anthocyanin transport proteins (Conn et al., 2008). Additionally, the extensibility of plant cell walls is an important mechanism for the regulation of salt tolerance (Boucaud et al., 1987; Nicol et al., 1998).

**FIGURE 12:**
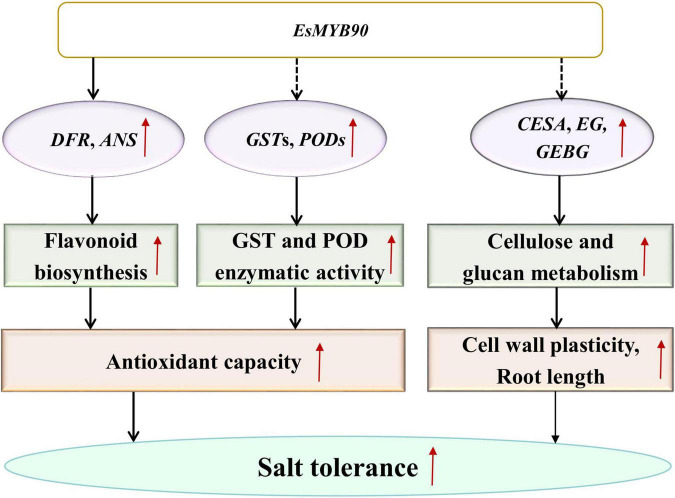
A proposed model for the regulation mechanism of *EsMYB90* in transgenic wheat. MYB, v-myb avian myeloblastosis viral oncogene homolog; GST, glutathione S-transferase; POD, peroxidase; *CESA*, cellulose synthase encoding genes; *GEBG*, glucan endo-1,3-β-glucosidase encoding genes; EG, endoglucanase encoding genes.

*Eutrema salsugineum* (salt cress), a halophyte model system, was proposed to exist for various protective mechanisms against the damage caused by salt stress (Inan et al., 2004; Taji et al., 2004; Gong et al., 2005; Zhang et al., 2013; Monihan et al., 2019). However, the *Eutrema EsMYB90* gene involved in enhancing growth and antioxidant enzymes activities as well as reducing oxidative injury in transgenic wheat has not been previously reported. Herein, our transcriptome and qRT-PCR analysis documented that many *GST* and *POD* family members as well as some flavonoid biosynthesis genes in *EsMYB90* transgenic wheat showed remarkably upregulated expression compared with the WT under the treatment of 200 mM NaCl for 24 h. Furthermore, under 200 mM NaCl treatment for 10 days, the *EsMYB90* gene significantly induced the antioxidant enzyme activities of GST and POD and markedly reduced the degree of membrane lipid peroxidation and the injury extent of salt stress in transgenic wheat plants. Hence, it is inferred that *Eutrema EsMYB90* enhanced the growth and antioxidant capacity of transgenic wheat during salt stress, which is mainly attributed to increased antioxidant enzymes activity and flavonoid biosynthesis *via* promoting the expression of *GST*, *POD*, and flavonoid biosynthesis related genes modulated by *EsMYB90* gene, to further detoxify ROS (Figure 12).

### Salt Tolerance of Transgenic Wheat Extends the Function of EsMYB90 Beyond the Flavonoid Pathway

*Eutrema EsMYB90*, an R2R3-MYB gene that encodes a nuclear localized protein and acts as a transcriptional activator of flavonoid biosynthesis, was characterized in our previous study (Qi et al., 2020). *EsMYB90* was introduced into dicotyledon species of tobacco, which led to the markedly upregulated production of 46 flavonoid compounds including anthocyanin, proanthocyanidin (PA or condensed tannin), flavonol, flavone, flavonone, and isoflavone (Qi et al., 2020, 2021). However, there have been no studies reported on the ectopic expression of *EsMYB90* in wheat, an important monocotyledonous dietary crop that provides food for almost half of the human population. In our study, the *EsMYB90* transgenic wheat lines were documented to have a higher antioxidant capacity with stronger antioxidant activities of GST and POD and less H_2_O_2_ accumulation compared to wild type plants, both in the control and 200 mM NaCl salt treatment conditions (Figures 3, 4), which indicated an enhanced capacity of scavenging H_2_O_2_ radicals in transgenic wheat plants. Meanwhile, the ectopic overexpression of *EsMYB90* in wheat not only led to an increased root length and fresh weight but also no other growth stunting, accompanied by an almost unaffected chlorophyll level (Figure 2). Therefore, this research provides a new wheat germplasm candidate resource with a higher antioxidant level, and it is favorable for the growth of wheat in salinity soil.

In summary, the result provided a new insight for understanding the roles of the *Eutrema* EsMYB90 transcription factor in transgenic wheat’s response to salt stress and the extension of *EsMYB90* function beyond the flavonoids pathway (Figure 12).

## Conclusion

In conclusion, the ectopic overexpression of *EsMYB90* revealed that under salt stress the transgenic wheat exhibited higher antioxidant levels and ROS-scavenging capacities, and longer root lengths, as well as higher transcript levels of stress-responsive genes such as *GST* and *POD* in the transgenic wheat plants, compared to the WT. Moreover, EsMYB90 protein could operate as a positive transcriptional regulator *via* binding directly to the MYB-binding elements of *pTaANS2* and *pTaDFR1* activating the transcription of *TaANS2* and *TaDFR1* genes, which will enhance the accumulation of flavonoid that functions in non-enzymatic antioxidant in transgenic wheat plants. This research provides a potential wheat germplasm resource that possesses a higher antioxidant capacity under salt stress, which extends our understanding of the roles of the EsMYB90 transcription factor in the responses of transgenic wheat plants to salt stress, offering an ideal candidate gene for crop improvement.

## Data Availability Statement

The original contributions presented in the study are publicly available. This data can be found here: https://www.ncbi.nlm.nih.gov/bioproject/PRJNA804652.

## Author Contributions

QZ conceived and designed the experiments and wrote the manuscript with contributions from CL, YZ, YQ, CD, and HZ. CL and YZ performed the experiments and data analysis. All authors have read and approved the final manuscript.

## Conflict of Interest

The authors declare that the research was conducted in the absence of any commercial or financial relationships that could be construed as a potential conflict of interest.

## Publisher’s Note

All claims expressed in this article are solely those of the authors and do not necessarily represent those of their affiliated organizations, or those of the publisher, the editors and the reviewers. Any product that may be evaluated in this article, or claim that may be made by its manufacturer, is not guaranteed or endorsed by the publisher.

## Footnotes

1
http://plants.ensembl.org/Triticum_aestivum/Info/Index

2
https://ftp.ncbi.nlm.nih.gov/blast/db/

3
http://geneontology.org/

4
http://www.genome.jp/kegg

5
http://bioinformatics.psb.ugent.be/webtools/plantcare/html/

## Supplementary Material

Supplementary Figure 1 Molecular identification of *EsMYB90* transgenic wheat plants by amplifying *3* × *Flag-EsMYB90* sequence. M: DL2000 DNA Marker; DDW, sterile double distilled water; WT, wild type; T1-4, T1-6, T1-7, T1-8, T1-9, and T1-30: different transgenic wheat lines; P: 3 × *Flag-EsMYB90-LGYOE3* plasmid used as PCR template.
