# Supplementary figures and images for: Eutrema EsMYB90 Gene Improves Growth and Antioxidant Capacity of Transgenic Wheat Under Salinity Stress
Source: Front Plant Sci. 2022 Apr 29;13:856163. doi: 10.3389/fpls.2022.856163 (PMC9102796; doi:10.3389/fpls.2022.856163)

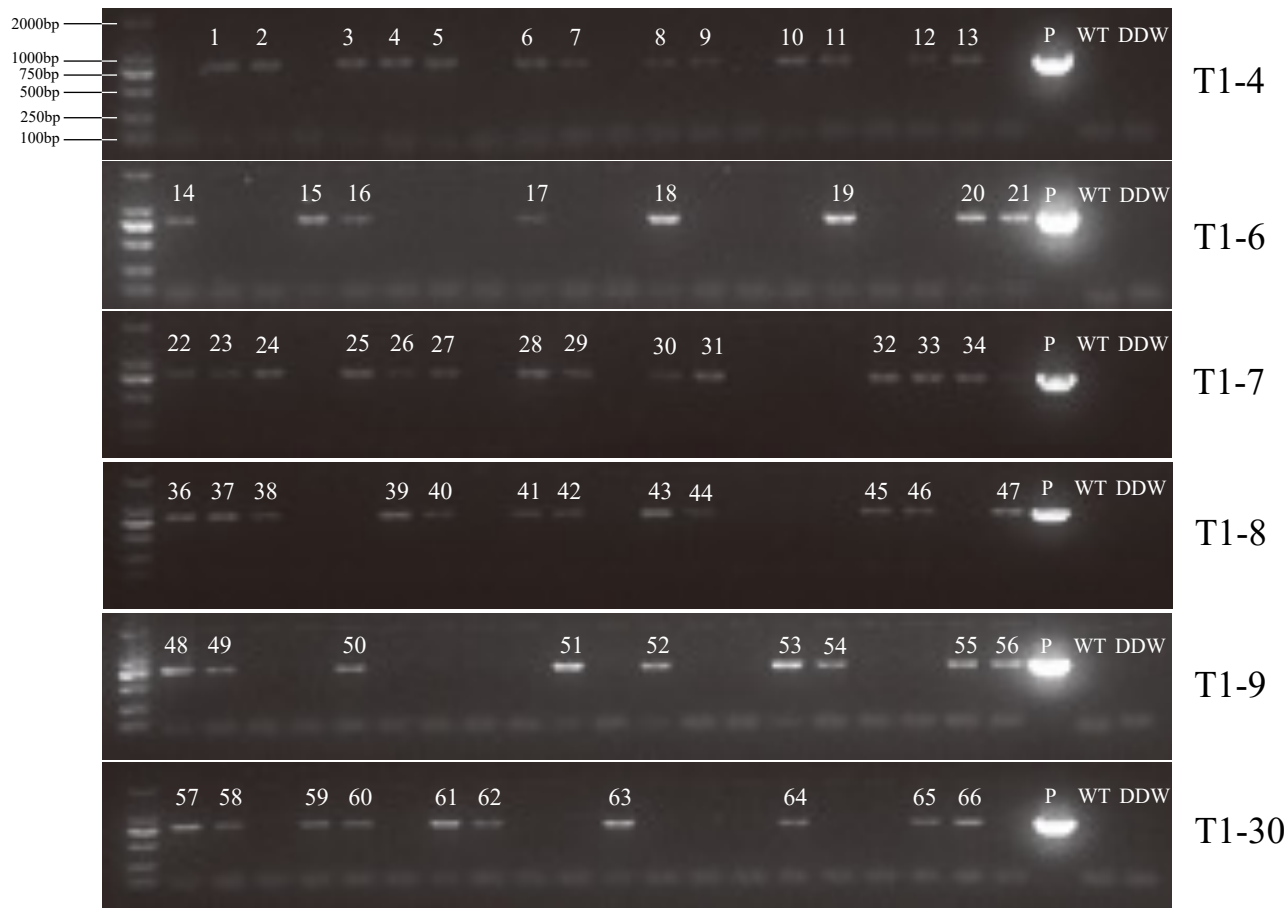

Supplement: Supplementary Figure 1 — Molecular identification of EsMYB90 transgenic wheat plants by amplifying 3 × Flag-EsMYB90 sequence. M: DL2000 DNA Marker; DDW, sterile double distilled water; WT, wild type; T1-4, T1-6, T1-7, T1-8, T1-9, and T1-30: different transgenic wheat lines; P: 3 × Flag-EsMYB90-LGYOE3 plasmid used as PCR template. [file Data_Sheet_1.PDF]
